# Perspectives of mental healthcare providers on pathways to improved employment for persons with mental disorders in two lower middle-income countries

**DOI:** 10.1186/s13033-020-00354-x

**Published:** 2020-03-30

**Authors:** Ikenna D. Ebuenyi, Barbara J. Regeer, Chinyere Aguocha, Joske F. G. Bunders-Aelen, Mònica Guxens

**Affiliations:** 1 Athena Institute, Amsterdam Public Health Research Institute, Vrije Universiteit Amsterdam, De Boelelaan 1085, 1081HV Amsterdam, The Netherlands; 2 ISGlobal, Hospital Clínic-Universitat de Barcelona, Barcelona, Spain; 3 Pompeu Fabra University, Barcelona, Spain; 4 Imo State University, Owerri, Imo state Nigeria; 5 Spanish Consortium for Research on Epidemiology and Public Health (CIBERESP), Instituto de Salud Carlos III, Madrid, Spain; 6 Department of Child and Adolescent Psychiatry/Psychology, Erasmus University Medical Centre–Sophia Children’s Hospital, Rotterdam, The Netherlands

**Keywords:** Healthcare providers, Work ability, Employment, Improved healthcare, Government commitment

## Abstract

**Background:**

Mental disorders affect employment and the ability to work, and mental healthcare providers are important in the promotion of health and employment for affected individuals. The objective of this study is to explore the perspectives of mental healthcare providers on pathways to improved employment for persons with mental disorders in two lower middle-income countries.

**Methods:**

Our study participants included mental healthcare providers (psychiatrists, occupational physicians, psychologists, and social care workers) from Kenya and Nigeria. Qualitative interviews and a focus group discussion were conducted with 15 professionals in Kenya and online questionnaires were completed by 80 professionals from Nigeria.

**Results:**

The study participants suggested that work is important for the recovery and wellbeing of persons with mental disorders. A complex interplay of factors related to the health of persons with mental disorders and the socioeconomic system in their setting were identified as barriers to their work ability and employment. Participants proposed four pathways to improved employment: including information on reducing stigma, better healthcare, policy advocacy in employment, and government commitment to healthcare and social welfare. Public education to reduce stigma and better healthcare were the highest reported facilitators of employment.

**Conclusions:**

Persons with mental disorders require multilevel support and care in obtaining and retaining employment. A better mental healthcare system is essential for the employment of persons with mental disorders.

## Background

At a global level, studies indicate that mental disorders adversely affects employment and the ability to work [1, 2], and the rate of employment is higher for those who are receiving treatment [3, 4]. Work ability refers to the extent to which a person is physically or mentally fit to meet the demands at the workplace [5], and it is affected by both common and severe mental disorders [3, 6]. It is pertinent to state that work and employment are different. While work includes all activities undertaken to produce goods and services for personal use or others, employment is a specific kind of work for pay or profit [7]. Hence, in this study work ability is different from employment; while the former refers to the person’s ability to work the latter refers working for pay or profit. Health professionals such as psychiatrists and psychologists are important in the promotion of work ability for persons with mental disorders [2, 8–10]. Their provision of care and support is pivotal to recovery and the ability to work [9, 11]. In the work environment, occupational health physicians and therapists also contribute to workers’ health and wellbeing by providing advice on mental health care and promotion [12–15]. A study using the Delphi method by Dekkers-Sánchez and colleagues suggest that occupational physicians and rehabilitations physicians have relevant role in work retention and return to work of employees [11].

Despite the suggestion in some studies that mental illness can lead to impaired social and occupational functioning [16], controversy and challenges remain in the certification of disability as it is sometimes keenly contested by society, persons with mental illness, and mental healthcare providers [15, 17]. In the United States, this has been a subject of several court cases, despite the guidance provided by the Americans with Disabilities Act [18]. In South Africa, persons with mental disorders still face an uphill task in obtaining the disability certificate they need to obtain social benefits [15]. In Kenya, the process is protracted and difficult [19], while in Nigeria, persons with mental illness are not regarded as living with a disability [20]. These challenges are barriers to mental health interventions in the workplace and integrated rehabilitation services that are essential for the employment of persons with a mental disorders [21, 22].

Health professionals are scarce and this is worse for mental health care [23]. In high-income countries, persons with mental illness mainly face demand-side barriers to health care [24]. Conversely, in low-income countries, both demand and supply-side barriers affect access to and the uptake of mental healthcare services [25, 26]. The 2014 Mental health Atlas Country profiles for Kenya and Nigeria indicated that there were 0.10 and 0.19 psychiatrists per 100,000 population respectively [27]. The funding for mental health care is less than 1% and 4% of the total health budget in Kenya and Nigeria respectively [28, 29]. In addition to the challenges in the health system, studies suggest that health professionals may have a negative attitude towards persons with mental illness [8, 30, 31], while also having an important part to play in their employment [8, 11, 30–32].

Despite the all-important role of mental healthcare providers in supporting the work ability of persons with mental disorders, we are unaware of any study in Africa that has explored the perspectives of mental healthcare providers and occupational physicians on the ability of persons with mental disorders to hold down a job. One study in Nigeria explored the attitude of doctors in general to persons with mental illness [8]. Our study takes this further by exploring specifically the perspectives of doctors involved in treating patients with mental disorders to identify pathways to improving their work ability. Improved work ability may lead to higher opportunities of employment. This study aims to explore the perspectives of mental healthcare providers on barriers and pathways to improved work ability and employment for persons with mental disorders in Kenya and Nigeria.

## Methods

### Study design and population

We set up two studies, one in Kenya and one in Nigeria, with different study designs: a qualitative study involving semi-structured interviews and a focus group discussion (FGD) in Kenya (thereafter referred to as Kenya Practitioners Study); and a quantitative study involving online questionnaires in Nigeria (thereafter referred to as Nigerian Practitioners Study). Given the dearth of empirical research on barriers and pathways to improved work ability and employment for persons with mental disorder in Sub Saharan Africa, it was anticipated that combining both studies in one research paper would add value as the qualitative and quantitative findings may allow for a deeper understanding of the issue in Lower Middle-Income Countries. The Kenya Practitioners Study involved mental health professionals (psychiatrists, psychologists, and social care workers) purposively selected from the Department of Psychiatry at the University of Nairobi Teaching Hospital in Kenya. Fifteen mental health professionals were involved in the interviews (n = 10) and one FGD (n = 5). The participants were selected based on their professional experience and some of them also worked and provided mental health services at Mathare Referral Hospital, which is the major in-patient public mental health hospital in Kenya. The Nigerian Practitioners Study used an online questionnaire [33] which was shared with psychiatrists and occupational/community health physicians in Nigeria. The questionnaire was shared through the social media platforms (WhatsApp and Facebook) of the Nigerian Medical Association and National Association of Resident Doctors. A total of 80 participants completed the questionnaire. Kenya and Nigeria are similar in terms of the unmet needs for mental health care (MHGAP) and in this study both settings were used to elicit views on the employability of persons with mental disorders in low-income settings [34]. This study is exploratory and part of a larger PhD study that sought to understand the barriers to and facilitators of employability for persons with mental disorders [35].

### Data collection

In the Kenya Practitioners Study, the qualitative data collection involved in-depth interviews which explored: perspectives on the ability to work of persons with mental disorders; perceived barriers to employment of persons with mental disorders; and perceived facilitators of employment for persons with mental disorders. The mental health professionals were encouraged to identify the common characteristics of those of their patients who were employed and what they thought would improve employment opportunities for those who had no job. The interviews lasted between 30 min and an hour and were conducted by the primary author IDE and an intern master’s student in public health. One FGD was conducted with five mental health professionals and was facilitated by IDE. The duration of the Focus Group was of approximately 90 min duration and aimed to validate and enrich the findings from interviews. When data saturation [36] was achieved, no further participants were invited for the interviews or FGD. The interviews and FGD were recorded and transcribed verbatim. The participants were provided with study information and their consent was also obtained.

In the Nigeria Practitioners Study, the online questionnaire was researcher-designed using Google Form. It consisted of seven open-ended and 11 closed questions in English. The questions for the qualitative and quantitative data were developed based on a literature review and empirical study by the authors [3, 37] It explored the perspectives of psychiatrists and occupational/community health physicians on the ability of persons with mental disorders to work and perceived barriers to and facilitators of their employment. The questionnaire also explored the pre-employment assessment of mental illness, accommodation made for persons with mental illness at the workplace, and what employers can do to improve employment for persons with mental disorders. For logistical reasons, we were unable to conduct qualitative interviews with mental health providers in Nigeria. The study information was provided, and informed consent was obtained before participants completed the survey.

In this study, we sought to understand the perspectives of mental health providers on the work ability of persons with both common and severe mental disorders. The interviews, FGDs, and questionnaire sought their opinion on the factors related with work ability, achieving and retaining employment for patients with mental disorders, without focusing on the stage of the disorder (i.e. before, during, or after treatment). Although common and severe mental disorders have different implications for both work ability and disability, we set out in our study to include the opinion of the healthcare providers on persons with all forms of mental disorders.

### Data analysis

In the Kenya Practitioners Study, the qualitative data was imported into Atlas.ti version 8 and analysed thematically [38]. Data was independently coded by IDE and CA and discussed with MG, BJR, and JFB. The differences were discussed after which the final themes emerged.

In the Nigeria Practitioners Study, the quantitative analysis, descriptive statistics were used to explore the socio-demographic characteristics of the healthcare providers, and their perceived barriers to and facilitators of employment and work ability for persons with mental disorders. The analysis was conducted using IBM SPSS version 23 (IBM, New York).

## Results

### Kenya Practitioners Study

#### Socio-demographic characteristics of study participants

Of the 15 participants in the Kenya Practitioners Study, there were four psychiatrists, nine psychologists, and two social workers.

#### Perspectives on the ability to work of persons with mental disorders

The mental healthcare providers involved in the interviews and FGD in the Kenya Practitioners Study, stated that they consider mental illness as a source of disability and that persons with mental illness can and should work. In addition, they revealed that in their clinical practice, they routinely encourage them to work when they are clinically stable because they considered it useful for recovery. One study participant stated that *‘I do very much try to encourage the patients to try to regain their original levels of functioning and to try as much as possible to maintain their jobs’* (Healthcare provider 4_Psychiatrist). However, they also stated that not all persons are able to work because of specific individual challenges. This idea was captured in a statement made by one study participant who declared that ‘*There are ones I have seen who are employed and others are who are not employed because of different reasons.* (Healthcare provider 2_Psychologist).

#### Perceived barriers to employment of persons with mental disorders

The healthcare providers in the Kenya Practitioners Study identified various interrelated barriers to the employment of persons with mental disorders, of which five major clusters were mental illness, dysfunctional health system, social stigma, socioeconomic status, and lack of government or policy commitment.

##### Mental illness

The mental healthcare providers suggested that factors related to the peculiar nature of the mental illness were a major barrier to employment and ability of persons with mental illness to work. Given the recurrent nature of mental illness and its associated impairment in social and occupational functioning, it was seen by respondents to limit the motivation of individuals with mental illness to work. This limitation was also perceived to affect those who were on treatment and suffering the side-effects of psychiatric medication. The illness and the effects of the medication were perceived to be related to the lack of interest or ability to work among persons with mental disorders.

*When you are given some of the drugs you sleep and you cannot function so that one causes another issue of utilizing both mental health facility and looking for employment among people who are mentally sick.* (Health Care Provider 3_Psychatrist)

It was noted that both the illness and the required treatment were perceived to have the same impact regarding employment. The side-effects of medication were considered to lead to non-compliance, which worsens the effects of the illness and further reduces functionality or work ability.

##### Dysfunctional health system

All mental healthcare providers agreed that mental health care is neglected and that the system is sub-optimal, manifested in the paucity of mental healthcare services, unavailable and expensive essential medication, and lack of insurance for mental health. According to the respondents, the lack of available mental health care means there are very few psychiatrists and psychologists, especially in rural areas. In addition, because there is only one in-patient mental health service in Kenya, people tend to it avoid because of stigma, and so prefer a private health service they cannot sustain in view of the chronic nature of mental illness.

*Our public mental health hospitals are few and people avoid them because there are so many patients within public hospitals and the resources are less staff who are overwhelmed; so they may not offer friendly services and have little time for the patients*… (FGD _Psychologist)

The problem of medication was also highlighted. Even for patients who choose to go to private hospitals and have private health insurance, the lack of essential medicines in the hospitals forces them to still make out-of-pocket payments for medication. The healthcare providers also noted that the few available medications in the public hospitals have a huge spectrum of side-effects which indirectly affects a person’s ability to work.

*I think access to medication is a problem but is no longer such a big problem…the bigger barrier is lack of purchasing power which leads to inability to buy medications and adherence to treatment. (*Healthcare provider 9_Psychologist)

These medication challenges are also related to unwillingness of insurance companies to offer cover to persons with a mental illness because it is perceived as a chronic illness, a pre-existing illness or related to alcohol use.

*I think poor health care utilization is related to finance because health care insurance does not cover mental illness… Insurance companies are still discriminative of persons with mental illness.* (Healthcare provider 3_Psychiatrist)

##### Social stigma

The study participants suggested that widespread stigma against persons with mental illness may have implications for their ability to work and employment opportunities. According to them, the stigma emanates from the family, community, employers and mental healthcare providers. This heightened stigma is perceived to be related to cultural perceptions and lack of understanding of mental illness leads to social isolation and exclusion.

*So, the society feels like you are not one of us and therefore you are not accepted among us, it’s like you are an outcast. So, because of that definitely one is stigmatized, and you cannot be allowed even to work in our[midst]… even the government itself does not allow many of these people, it does not employ them.* (Healthcare provider 1_Psychologist)

The health care providers suggested that stigma is also encountered among employers who may fire an individual on the grounds even of common mental health disorders such as anxiety.

*So, I think the biggest barrier really in addition to money is the stigma around mental health. …. you don’t even have to be psychotic, having something as simple as anxiety, depression can get you fired and you are never getting a job again.* (Healthcare provider 10_Psychologist)

The stigma is not restricted to employers or social perceptions, but also happens in hospitals among the healthcare providers charged with caring for patients and improving their ability to work. This was aptly captured by one of the study participants: ‘*People with mental illness go through a lot of stigma that stops them from seeking care or going to school…. And even when they come to seek care, they would receive stigmatization from the people treating them…’* (Healthcare provider 9_Psychologist).

In the Kenya Practitioners Study, the respondents said it was uncommon to make a pre-employment assessment of mental illness on account of stigma. They also suggested that they do not advise their patients to disclose their mental health status because of the likelihood of stigma. A psychiatrist stated: *We don’t encourage them to fully disclose, yeah. We think they need to be strategic about… because most of the time it would jeopardize their job opportunity (*Health care provider 4_Psychiatrist).

##### Low socioeconomic status

The healthcare providers suggested that critical to employment is the socioeconomic status of the individual, since this determines whether the individual is educated, has the required skills to qualify for employment and the money to set up their own business. One study participant stated: ‘*For me, there is a correlation between socioeconomic status and work ability, because if you are not able to access good health care, meaning even the treatment will be poor, meaning even for you to work is also a challenge…* (Health care provider 7_Social worker).

This point is further elaborated by the study participants in their comparisons of their patients who are foreign or have a higher socioeconomic status and those who have a low socioeconomic status. According to them, patients with a higher socioeconomic status do not face the challenges of securing or retaining job and this may be related to the stability of their illness, ability to obtain care and often higher educational level, all of which are also useful for employment and ability to work.

*Those who are poor let’s say those who are quite poor most probably you are not going to get even that employment, and then that means probably you will become poorer. But those who are a little bit wealthy, I think they have better services, they are given better services even in the hospitals because they are able to access the best psychiatrist for example and they get proper medication. And I think also those who are wealthy also kind of look at it as an illness, it’s not just like, it’s not something that people don’t understand what it is.* (Healthcare provider 1_Psychologist)

The predicament of people of low socioeconomic status was noted by participants to be like a vicious circle in which they are unable to help themselves. Another study participant declared that ‘*… money is an issue. You see these persons may go home and they have the medication. But one month later, there is no money to buy the medication so they are psychotic again and cannot work…’* (Healthcare provider 10_Psychologist).

##### Lack of government and policy commitment

Lack of government and policy commitment was identified as a major limitation to work opportunities for persons with mental disorders because of its relationship to health care and social welfare. According to the respondents, government apathy may explain the failure to implement policies and healthcare commitments, the absence of enforcement mechanisms to ensure inclusive employment and discriminatory health insurance scheme, and the lack of functional essential medicines in public hospitals. The mental healthcare providers suggested that government commitment and investment in mental health care is poor.

*We are fighting for health from all angles and that is a big challenge not only for mental health sector. Budgetary allocation for health itself is bare minimum now start thinking mental health…* (Healthcare provider 5_Psychiatrist)

This low investment in health has implications for access to or uptake of mental health care, which also affects the ability to work of persons with mental disorders. According to them, this lack of commitment is evident in the dysfunctional mental healthcare services, which makes it possible for only the middle class with more disposable income to obtain mental health care and health care in general. They further highlighted the difficulties of managing mental health care without health insurance.

*….they don’t have insurance they don’t have the money then they don’t come for clinic they don’t come for follow up and yeah so they are at a disadvantage.* (Healthcare provider 5_Psychiatrist)

The government and related factors interact with problems in the work and social environment to influence employment opportunities for persons with mental disorders.

#### Perceived facilitators of employment for persons with mental disorders

In the Kenya Practitioners Study, the mental healthcare providers suggested that greater employment for persons with mental disorders is possible through the combination of several factors acting together. Four clusters of factors were identified including information on stigma reduction, improved health care, policy advocacy on employment, and government commitment to health care and social welfare.

##### Information of stigma reduction

The healthcare providers suggested that providing relevant information on mental illness would help reduce stigma against persons with mental illness. This information would be aimed at individuals in society, employers, mental healthcare providers, and policy-makers. The expected ripple effect of information and relation to improved employment is evident in the words of one of the study participants who declared:

*If stigma is reduced and even employers are able to understand about mental illness that when they are stable they are able to work; when they are sick, they get treatment, I think that could help. If employers are understanding[about] of mental conditions, that would enable them to recover.* (Healthcare provider 9_Psychologist)

The critical nature of educating society about mental illness is further captured in the statement: *‘…So I think first we need to educate the society to know about mental illnesses. That is key so …that people would feel free to seek employment. Because there are those who are sick but they are afraid of going to seek employment and there are those who are not even ready even to disclose because of the fear. So, first is sensitizing the society to know that this illness is like any other illness’* (Healthcare provider 1_Psychologist).

##### Improved health care

The healthcare providers advocated improved health care as a way to enhance the ability of persons with mental disorders to find work. They suggested that a better health system would ensure the availability of optimal and affordable care, which would indirectly enhance adherence to treatment and ensure a stable clinical state that is relevant for work ability.

*Health institutions and professionalism are also key; so that people can access care and also in a non*-*judgmental manner….we have very few facilities around which deal with mental health like for example now we have only Mathare, so we need more of those kind of facilities. We need also even rehabilitation centres…* (Healthcare provider 1_Psychologist).

The theme of advocacy for improved care and professionalism was also closely related to the need of healthcare providers to protect patients and cater for their health needs. One participant stated: *‘Part of advocacy is protecting the patient. So, we cannot say that we are advocating for improvement to health care while not protecting the patient’* (Health care provider 10_ Psychologist).

##### Policy advocacy on employment

In the Kenya Practitioners Study, the study participants identified the importance of a supportive work environment and suggested that practical policies to improve reasonable accommodation would enhance both employment and retention in jobs for persons with mental illness. They advocated affirmative action in the employment of persons with mental illness and close collaboration with employers to ensure that disclosure does not lead to stigma but aids in the provision of workplace accommodation: ‘*…with support, if these people can be supported then they are able to progress but when they are in an environment where people do not understand what is all about mental illness…’* (Healthcare provider 7_Social worker).

The mental healthcare providers advocated for local companies to emulate the employment polices of international companies that not only employ persons with mental disorders but also provide them with mental healthcare packages.

*And because they don’t know what to do with you they would rather not employ you so that is further stigmatization they are experiencing. But the international companies and institutions I think they kind of understand this kind of condition. Like for example I have dealt with a number of international kind of institutions and there are a number of the patients they brought me or clients who have been in war*-*torn areas where there is a lot of fighting and majority of them have post*-*traumatic stress disorder. So when they come they actually bring them for help and without the intention of sacking them after treatment. But then the local companies sometimes are very shallow understanding about even how to deal with these people.* (Healthcare provider 1_Psychologist)

In order for employers to provide reasonable accommodation in the workplace, some participants recommended that disclosure might be useful in order to for employers to help employees with mental disorders. One of the participants stated: *‘I think disclosure to people who are your support is important; I think it’s a must’* (Healthcare provider 1_Psychologist). It is pertinent to note that this suggestion to disclose was perceived as advisable only after the individual has gained employment, as stated earlier.

##### Government commitment to health care and social welfare

In the Kenya Practitioners Study, government commitment to health care and welfare was considered central and related to most of the factors that are relevant to greater employment of persons with mental disorders. According to the participants, this is because government commitment would ensure that all the limitations in the health system, including addressing the shortage of mental health professionals, non-availability of essential mental health medications and rehabilitation services, and discriminatory health insurance practices. This crucial role of the government is captured in the statement:

*…first of all, the government needs to see mental illness as something that needs to be addressed by coming up with a mental health policy and integrating into it ways of implementation. I think that can improve a lot, can improve first of all inpatient facilities so that people would stop stigmatizing against Mathare, so in terms of employing people, getting enough beds, getting drugs and also subsidizing the medication.* (Healthcare provider 6_Social worker)

The role of government in addressing the needs of persons with social disadvantages who also face challenges of the health care and education that are critical for employment is also highlighted in the statements of the study participants:

*I think government has a very, very big role I mean for a long, long time health has been neglected in low*-*resource or developing countries context; however, you want to call it. The fact that the budget is great on security and less on primary education or health in itself very telling and we need to come up with institutional mechanisms to address poverty and socioeconomic disadvantages.* (Healthcare provider 9_Psychologist)

This statement is relevant because the healthcare providers identified self-employment as a useful alternative to formal employment, which is scarce. Self-employment or self-help businesses offer persons with mental disorders the flexibility and control that is lacking in formal jobs.

*…what I have noticed is that there are more of those who are self*-*employed than those who are formally employed are basically again what we’ve seen that securing employment is not easy for them so majority just choose to have some private business or something somewhere just to employ themselves, yes.* (Healthcare provider 1_Psychologist)

Achieving self-employment may be dependent on dependent on financial resources and government social welfare programmes, because of the social disadvantages facing most persons with mental illness from very early in life.

*I think they have social protection factors like being educated, being employable because of that educational training or skill that they have, often times is high level of management positions so there is already some success and some sort of problems that they have learnt and they are managing to the relationships or inciting in stress through psychotherapy and kind of psychopharmacologies is not very difficult*. (Healthcare provider 9_Psychologist)

### Nigerian Practitioners Study

#### Socio-demographic characteristics of study participants

Table 1 outlines the socio-demographic characteristics of the study participants. Of the 80 healthcare providers, 43 were psychiatrists, 11 were occupational health physicians, 19 were community health physicians while seven did not state their profession. Their mean years of practice was 8.7; and 37.5% had practised for more than 10 years.

**Table 1:** Socio-demographic characteristics of healthcare providers from the Nigeria Practitioners Study

Variable	Category	Distribution^a^
Sex	Female	28 (35.4)
	Male	51 (64.6)
	Missing	1
Age (years)		37.9; 37; 27–64
Profession	Psychiatrist	43 (53.8)
	Occupational health physician	11 (13.8)
	Community health physician	19 (23.8)
	Other	7 (8.8)
Fellowship status	Completed residency	31 (38.8)
	Currently doing residency	45 (56.3)
	Not applicable	4 (5.0)
Years of practice	Years in continuous	8.7; 7; 3–27
	Less than 10 years	49 (61.3)
	More than 10 years	30 (37.5)
	Missing	1

^a^Values are N (percentage) for categorical variables and mean; median; range for continuous variables

#### Perspectives on ability to work of persons with mental disorders

In the Nigerian Practitioners Study, 96.2% of the healthcare providers believed that persons with mental disorders can work and 76.3% said they have persons with mental disorders at their workplace (Table 2). When asked whether they thought such persons posed a risk at the workplace, 91.3% said sometimes and 7.5% said never. The majority (86.3%) of the healthcare providers felt they had a role in enhancing job opportunities for persons with mental disorders. In response to what they can do to enhance job opportunities for persons with mental disorders, majority of the healthcare providers from Nigeria suggested early intervention and rehabilitation (39.1%) and public education to reduce stigma (25.0%) of mental illness. When asked to suggest conditions under which persons with mental disorders can work, the responses ranged from: under supervision (27.8%), when clinically stable (26.4%), when on treatment or medication (16.7%), unsupervised like other people (11.1%), when they want and have the capacity to work (8.3%), and when they have the capacity to work in a sheltered setting (1.4%).

**Table 2:** Perspectives on ability to work of persons with mental disorders from the Nigeria Practitioners Study

Variable	Category	Distribution N (%)
Do you have persons with mental disorders at your workplace?	No	4 (5.0)
	Yes	61 (76.3)
	I do not know	15 (18.8)
Do you think persons with mental disorders can work?	No	3 (3.8)
	Yes	75 (96.2)
	Missing	2
Do you think persons with mental disorders pose a risk at work?	Always	1 (1.3)
	Never	6 (7.5)
	Sometimes	73 (91.3)
Are there things you can do to enhance jobs for persons with mental disorders?	No	11 (13.8)
	Yes	69 (86.3)
What are things you can do to enhance job opportunities for persons with mental disorders?	Public education to reduce stigma of mental illness	16 (25.0)
	Early intervention and rehabilitation	25 (39.1)
	Demonstrating ability to work of persons with mental disorders	4 (6.3)
	Supportive work environment	6 (9.4)
	Policy advocacy and affirmative action	4 (6.3)
	Education of employers	3 (4.7)
	Others	6 (9.4)
	Missing	16
Under what conditions should persons with mental illness be allowed to work?	Under supervision	20 (27.8)
	When clinically stable	19 (26.4)
	When on treatment or medication	12 (16.7)
	Unsupervised like others	8 (11.1)
	In sheltered work settings	1 (1.4)
	When they want and have the capacity to do the job	6 (8.3)
	Others	6 (8.3)
	Missing	8

In the questionnaire, the term mental disability was used to refer to mental disorder

#### Perceived barriers and facilitators to employment of persons with mental disorders

Table 3 shows that 11.3% of the participants of the Nigerian Practitioners Study reported that pre-employment assessment of mental illness affected employment opportunities for persons with mental illness; yet it was also perceived as one of the reasons for the absence of workplace accommodation. The majority of respondents (77.3%) said that pre-employment assessment of mental illness was not applicable in their workplace. Although 76.3% of the participants in the Nigerian Practitioners Study reported that they have persons with mental illness in their workplace, only 15% have made the relevant workplace accommodation.

**Table 3:** Perceived barriers and facilitators to employment of persons with mental disorders from the Nigeria Practitioners Study

Variable	Category	Distribution N (%)
Do you have pre-employment assessment at work?	No	71 (88.8)
	Yes	9 (11.3)
How often has pre-employment assessment affected opportunities at your workplace?	Never	8 (10.7)
	Not applicable	58 (77.3)
	Sometimes	9 (12.0)
	Missing	5
Is there workplace accommodation at your workplace?	No	68 (85.0)
	Yes	12 (15.0)
What factors can enhance the job opportunities for persons with a mental disorder?	Public education to reduce stigma of mental illness	21 (27.3)
	Early diagnosis and treatment	18 (23.4)
	Improved health care for persons with mental disorders	6 (7.8)
	Supportive work environment	8 (10.4)
	Family support	2 (2.6)
	Policy advocacy and affirmative action	9 (11.7)
	Formal education and training for persons with mental disorders	7 (9.1)
	Other	6 (7.8)
	Missing	3
Why is there no accommodation for persons with a mental disorder at your workplace?	I don’t know	9 (20.5)
	No pre-employment assessment	1 (2.3)
	No provisions by employer/management	9 (20.5)
	Lack of awareness of its usefulness	5 (11.4)
	Non-disclosure of mental illness at employment	2 (4.5)
	Financial constraints	3 (6.8)
	Neglect of mental illness	5 (11.4)
	Other	10 (22.7)
	Missing	36

In the questionnaire, the term mental disability was used to refer to mental disorder

Public education to reduce stigma was the highest (27.3%) reported factor that could improve job opportunities for persons with a mental disorders while early diagnosis and treatment of mental illness was the second-highest (23.4%) reported factor to enhance ability of persons with mental illness to work (Table 3). Also, 7.8% of the healthcare providers reported that improved health care was relevant for job opportunities for persons with mental disorders. In addition, supportive work environment (10.4%), policy advocacy and affirmative actions (11.7%) and formal education and training for persons with mental disorders (9.1%) were identified as factors that can enhance job opportunities for persons with mental disorders (Table 3)

## Discussion

Our study is the first in Africa to explore the perspectives of mental healthcare providers on employment of persons with mental disorders. In Kenya, we identified the impact of mental illness, a dysfunctional health system, social stigma, low socioeconomic status, and lack of government or policy commitment as the major barriers to employment of persons with mental disorders, while public education on reducing stigma, improved mental health care and policy advocacy on employment are some of the suggested facilitators of employment. In Nigeria, public education to reduce stigma and improved health care were the highest reported facilitators of employment.

In identifying the pathways to employment for persons with mental disorders we explored the perspectives of healthcare providers on their work ability. The majority of the study participants in both studies identified the relevance of work for persons with mental disorders and its role in the recovery process. The relevance of this finding has been previously recorded [39, 40]. Mental healthcare providers who support employment for persons with mental illness in these settings offer both care and psycho-education. It is, however, pertinent to note their observation that not all persons with mental disorders can work. We believe that this does not suggest a negative view on the ability of persons with mental disorders to work, but rather a declaration of the realities of their perception of diverse abilities. This observation is relevant and needs to be taken into consideration by programmes that promote employment so that a ‘one-size-fits-all’ approach is not adopted but considerations and relevant provisions made for those who may have different needs.

We identified five perceived barriers to employment namely: mental illness, dysfunctional health system, social stigma, low socioeconomic status, and lack of government or policy commitment. The impact of mental illness itself is a known barrier to employment and its capacity to affect social and occupational functioning has been documented [16]. Our study identified the dysfunctional health system as another barrier. The complexity and extent of the problems in the health systems are wide and merit attention. The shortage of mental-health professionals and lack of essential medication for mental health are pivotal for mental health care [23, 41]. The challenges of healthcare financing and discriminatory practices of health insurance companies compound the difficulties faced by persons with mental illness, particularly if they also face socioeconomic challenges. The essential nature of effective health systems are evident in the variations across countries depending on commitment to healthcare financing and effective health care [42] and the lack of health insurance is associated with high unmet need for mental health services [43]. The widespread social stigma that cuts across all social strata in this study is another barrier. The fear and lack of information among employers reinforces the stigma and limits their employment of persons with mental disorders [44]. The stigma borne by the family and other individuals deprives persons with mental disorders of the health care they deserve; and, sadly, the actions of certain healthcare workers may also stigmatize them further. Stigma and discrimination thrive on cultural bias and ignorance and constitute a limitation to work opportunities for persons with mental disorders [3, 31, 45]. Studies have previously documented the interplay between poverty and mental health [41, 46]. Another barrier to the employment of persons with mental disorders identified in this study is the lack of government or policy commitment to mental health care. This finding places the onus of responsibility on government agencies. The socio-economic problems faced by persons with mental disorders may worsen their experience of Social drift in addition to their unemployment challenges [41, 47].

Our study also confirms the need for policy reform in order to improve employment opportunities for persons with mental disorders. The four characteristics of these pathways include information on stigma reduction, better health care, policy advocacy in employment, and government commitment to health care and social welfare. In order to address the ignorance and misinformation regarding mental disorders, there is need to address the issue of stigma. This might ensure that employers better understand the ability of persons with mental disorders to work and the need to provide workplace support. It could also enhance social and family support for persons with mental disorders. Addressing stigma may also help in enhancing the quality of mental healthcare services [8, 9, 30]. We believe that reduction of stigma may lead to the improved quality of mental healthcare services, which in turn may change discriminatory insurance policies and effective procurement of essential medicines in public hospitals and clinics. Studies have suggested that institutionalized stigma in the health system affects access to and uptake of care [47, 48].

Our study also suggests the implementation of policy advocacy on employment to improve employment opportunities for persons with mental disorders. This would ensure that mechanisms that guard against discriminatory employment practices are in place and that dialogue and collaboration with employers is used to achieve fair employment practices. There is evidence in support of this finding and studies suggest that adoption of inclusive employment policies facilitates employment for persons with disorders [40, 49]. Lastly, in order to improve employment opportunities for persons with mental disorders, government commitment to health care and social welfare is critical. Health care, education, and social welfare are human rights and when governments create the environment in which they flourish, these enhance social participation and inclusion [24, 42, 50]. The importance of universal health coverage cannot be overemphasized and Goal 3 of the Sustainable Development Goals (SDGs) supports an increase in health financing to ensure health care for all [51]. This may be achievable only when governments improve health financing and especially mental health care. Social welfare would ensure rehabilitation services and there is evidence to suggest that cash transfers and loans may enhance self-employment for persons with disorders in low-income settings [41].

Our study also identified certain issues that were perceived as both barriers and facilitators of employment for persons with mental disorders. Pre-employment assessment and disclosure were seen as both barriers to and facilitators of employment. Although pre-employment assessment provides information on the needs of an employee in terms of making reasonable accommodation, it may also be a limitation to employment [2]. This observation has been made by other studies and is a vexed issue similar to perceptions about disclosure. The argument for disclosure of mental illness to employers is that it may foster the provision of reasonable accommodation and is also relevant to enable employers to access the tax rebates and benefits they obtain from the government for employing persons with disorders. Studies also suggest, however, that disclosure of a mental disorder may end a job interview and lead to stigma at the workplace [2].

By utilizing both qualitative and quantitative methods, this study offered an opportunity to explore the observations of mental healthcare providers and their contextual experiences. Collecting data from two lower middle-income countries such as Kenya and Nigeria enabled us to understand the perspectives of mental health professionals in these settings. The findings from the study in Kenya were corroborated by the results of the study in Nigeria and indicated the similarities in employment challenges faced by persons with mental disorders in low-income contexts.

This study also has certain limitations. The participants in the qualitative study were purposively selected and may have introduced a form of selection bias that affects the generalization of our study findings. Our efforts to include psychiatrists in the FGD conducted in Kenya were unsuccessful, and only psychologists were finally included. The reports of the healthcare providers involved in the Nigerian study may also have been affected by recall bias or social conformity. For logistical reasons, we were unable to conduct qualitative interviews with mental health professionals in Nigeria. Therefore, a direct comparison of the results from both countries was not possible. However, Kenya and Nigeria are similar in terms of the unmet needs for mental health care (MHGAP) and in this study both were used to elicit views on employment of persons with mental illness in low-income settings [34]. We acknowledge our study may be limited in its coverage of only Kenya and Nigeria. Nigeria is larger than Kenya and it was reported in 2008 to have 1.7 times more mental health professionals than Kenya [34]. However a recent mental health survey in Nigeria from January 2020 suggests that many mental health professionals have left Nigeria on account of brain drain, thereby making several countries in Africa (e.g. South Africa, Egypt, and Kenya) better resourced in regard to mental health personnel [28].

Also, we could not confirm the profession of about 8.8% of the respondents who completed the online questionnaire. Nevertheless, we included their reported information on the perspectives on employment of persons with mental disorders and on the barriers and facilitators to avoid selection bias.

## Conclusion

The right to work is a human right and crucial for the recovery of persons with mental disorders [39, 52]. Mental healthcare providers play a crucial role in the employment of persons with mental disorders However, a complex interaction of factors limits employment opportunities for such persons. In Kenya, the identified barriers to the employment of persons with mental disorders include the impact of mental illness itself, a dysfunctional health system, social stigma, low socioeconomic status and lack of government or policy commitment. Public education on reducing stigma, better mental health care and policy advocacy on employment are some of the suggested facilitators of employment. In Nigeria we found that public education to reduce stigma and improved health care were identified as key facilitators of work ability. These factors are closely interconnected and require a systemic approach. An improved healthcare system and government commitment to mental health care and social welfare are essential for the employment of persons with mental disorders.

## Data Availability

Anonymized data are available upon request from researchers, who meet the criteria set out in the Vrije Universiteit, Amsterdam data policy. Request for data may be made through the corresponding author.
